# Broad-Spectrum Antiviral Activity of the Amphibian Antimicrobial Peptide Temporin L and Its Analogs

**DOI:** 10.3390/ijms23042060

**Published:** 2022-02-13

**Authors:** Carla Zannella, Annalisa Chianese, Luciana Palomba, Maria Elena Marcocci, Rosa Bellavita, Francesco Merlino, Paolo Grieco, Veronica Folliero, Anna De Filippis, Marialuisa Mangoni, Lucia Nencioni, Gianluigi Franci, Massimiliano Galdiero

**Affiliations:** 1 Department of Experimental Medicine, Università degli Studi della Campania Luigi Vanvitelli, 80138 Naples, Italy; carla.zannella@unicampania.it (C.Z.); annalisa.chianese@unicampania.it (A.C.); lucianapalomba88@gmail.com (L.P.); veronica.folliero@unicampania.it (V.F.); anna.defilippis@unicampania.it (A.D.F.); 2 Department of Public Health and Infectious Diseases, Laboratory Affiliated to Istituto Pasteur Italia—Fondazione Cenci Bolognetti, Sapienza University of Rome, 00161 Rome, Italy; mariaelena.marcocci@uniroma1.it (M.E.M.); lucia.nencioni@uniroma1.it (L.N.); 3 Department of Pharmacy, Università degli Studi di Napoli ‘Federico II’, 80131 Naples, Italy; rosa.bellavita@unina.it (R.B.); francesco.merlino@unina.it (F.M.); paolo.grieco@unina.it (P.G.); 4 Department of Biochemical Sciences, Laboratory Affiliated to Pasteur Italia—Fondazione Cenci Bolognetti, Sapienza University of Rome, 00185 Rome, Italy; marialuisa.mangoni@uniroma1.it; 5 Department of Medicine, Surgery and Dentistry “Scuola Medica Salernitana”, University of Salerno, 84081 Baronissi, Italy; gfranci@unisa.it

**Keywords:** antimicrobial peptides, AMPs, temporins, antiviral activity, virus-host interaction, HSV-1, SARS-CoV-2, frog peptides

## Abstract

The COVID-19 pandemic has evidenced the urgent need for the discovery of broad-spectrum antiviral therapies that could be deployed in the case of future emergence of novel viral threats, as well as to back up current therapeutic options in the case of drug resistance development. Most current antivirals are directed to inhibit specific viruses since these therapeutic molecules are designed to act on a specific viral target with the objective of interfering with a precise step in the replication cycle. Therefore, antimicrobial peptides (AMPs) have been identified as promising antiviral agents that could help to overcome this limitation and provide compounds able to act on more than a single viral family. We evaluated the antiviral activity of an amphibian peptide known for its strong antimicrobial activity against both Gram-positive and Gram-negative bacteria, namely Temporin L (TL). Previous studies have revealed that TL is endowed with widespread antimicrobial activity and possesses marked haemolytic activity. Therefore, we analyzed TL and a previously identified TL derivative (Pro^3^, DLeu^9^ TL, where glutamine at position 3 is replaced with proline, and the D-Leucine enantiomer is present at position 9) as well as its analogs, for their activity against a wide panel of viruses comprising enveloped, naked, DNA and RNA viruses. We report significant inhibition activity against herpesviruses, paramyxoviruses, influenza virus and coronaviruses, including SARS-CoV-2. Moreover, we further modified our best candidate by lipidation and demonstrated a highly reduced cytotoxicity with improved antiviral effect. Our results show a potent and selective antiviral activity of TL peptides, indicating that the novel lipidated temporin-based antiviral agents could prove to be useful additions to current drugs in combatting rising drug resistance and epidemic/pandemic emergencies.

## 1. Introduction

One of the biggest public health challenges is emerging viral infections due to possible epidemic and pandemic risks. Furthermore, widespread viral infections pose serious problems due to the possible onset of resistance to available antivirals. This is looming due to the limited number of therapeutic options available against many viruses. The current armamentarium available for antiviral drugs has significantly expanded in recent decades and currently encompasses several viral families [1,2,3]. The current COVID-19 pandemic, and also the previous emerging viral outbreaks (swine flu, Ebola, other coronaviruses, Nipah, Zika and others), highlight the urgent need to develop broad spectrum antivirals [4,5,6]. In practice, most antivirals are designed with the aim of blocking the function of a specific viral protein crucial for a precise mechanism in the replication cycle, so this target is likely unique to a specific virus or viral family. In fact, the specific characteristics and peculiarities of the replication of each viral family act as an obstacle to the realization of broad-spectrum antiviral agents. Moreover, since viruses use the functional apparatus of the host cell for most of their activity, the number of putative direct antiviral targets is further reduced. In consideration of the current COVID-19 pandemic, it is unlikely that the traditional virus-specific paradigm of antiviral drug development can be implemented for the immediate availability of drugs, but it is essential to act promptly and effectively against new pathogens that cause unexpected but lethal infections. Within this perspective, the one-drug-to-one-target paradigm for antiviral drug discovery has proved inadequate for responding to an increasing diversity of viruses deadly in humans.

This underlines the urgency of designing broad-spectrum antivirals that can act on multiple viruses by intercepting some common steps of their life cycle rather than specific viral proteins [7]. In this context, it is important to promote the development of novel antiviral agents with a broad spectrum of activity based on alternative mechanisms of action.

Therapeutic peptides have become interesting tools in drug discovery, with antimicrobial peptides (AMPs) widely studied for their potential antiviral properties as evidenced by the wealth of data accumulated in recent years [8,9,10,11,12]. The study of the antiviral activity of AMPs has grown substantially in recent years to allow the compilation of a specific online database, such as the antiviral peptide (AVP) database (AVPdb—http://crdd.osdd.net/servers/avpdb/, accessed on 31 December 2021) [13,14]. Even though the number of AMPs endowed with antiviral activity is still low, they have shown enormous potential for being translated into pharmaceutically available antiviral drugs. AVPs can derive from natural sources, such as those isolated from mammals and insects, or obtained by artificial means using bioinformatic tools. Their principal mechanisms of action are conveyed by directly acting on the virus particle by a virucidal effect or competing for the binding site on the host cell membrane and interfering with their attachment and entry. However, AVPs may also act in further stages of the viral cycle, such as viral replication or viral egress (for an updated comprehensive review on AVPs see reference [15]). Of importance are the interactions of AVPs with biological membranes, which lead to modification of rigidity and curvature of the viral and/or cell membrane and consequent reduction of cell susceptibility to infection.

Several AMPs have been reported to be derived from skin secretions of different amphibian species, but relatively few frog-derived peptides with antiviral properties have been described in the literature. Frogs produce AMPs in dermal glands and release them onto the skin by a holocrine mechanism upon stress of physical injury [16,17,18]. Generally, these peptides have common features, such as a net positive charge (due to the presence of basic amino acids), the presence of at least 50% hydrophobic amino acids, and an amphipathic α-helical secondary structure with a length from 10 to 50 amino acids [19,20,21]. The most known amphibian AMPs with antiviral activity are the magainins from frog *Xenopus laevis*. Both magainin1 and 2 showed an efficient virucidal effect on viruses belonging to the *Herpesviridae* family, probably by means of interaction with the viral envelope components and subsequent disruption of the envelope integrity [22,23]. The antiviral activity of dermaseptins (produced by frogs from the Phyllomedusa genus) and their derivatives has been described against herpes simplex virus type 1 and 2 (HSV-1, HSV-2) [24,25], human immunodeficiency virus type 1 (HIV-1) [26], and rabies virus [27] via a virucidal mechanism of action disrupting viral envelopes, but also affecting the early stages of the intracellular infection. In vivo studies using mice showed a strong protective effect within the range of 100–200 μg with a 75% increased survival of animals after a challenge with rabies virus [27]. A further amphibian skin-derived AVP is HS-1 from *Hypsiboas semilineatus*, which is active against Dengue virus 2 and 3. Both viruses were The the Indian frog *Hydrophylax bahuvistara* produces a peptide, urumin, with strong inhibitory activity on influenza virus replication both in vivo and in vitro [28]. Interestingly, some influenza subtypes (H1N1, H1N2) were mainly affected compared to other subtypes (H3N1 and H3N2), pointing out a preferential interaction with hemoagglutinin 1 [29].

One of the largest family of amphibian peptides is represented by temporins, which were first isolated from the skin secretions of the European common red frog *Rana temporaria* [30,31]. Temporins are among the smallest AMPs known, with a length ranging from 10 to 14 amino acids, a weak cationic character due to the presence of few basic residues in their sequence, and with an amphipathic α-helical conformation in hydrophobic environments. They are mainly active against Gram-positive bacteria [17,32]. Some temporins have proved their efficacy as AVPs, such as temporin B (TB) with a virucidal activity against HSV-1. In fact, preincubation of HSV-1 purified virions with 20 µg/mL of TB led to a 5-log reduction of virus titers. By transmission electron microscopy, a clear disruption of the viral envelope was observed. Moreover, TB could also alter other stages of the HSV-1 life cycle, including the attachment and the entry of the virus into the susceptible host cell [33]. The isoform A of temporin has also shown the ability to inhibit virus infection by reducing the replication of the channel catfish virus and the frog virus 3 [34]. Most recently, a further temporin has been investigated for its antiviral activity. Temporin-SHa (SHa) and its (K3) SHa analog (with substitution of the serine in position 3 with a lysine [35] to increase its net positive charge while retaining the α-helical structure), have been described to significantly inhibit HSV-1 replication in human primary keratinocytes at micromolar concentrations [36]. Finally, temporin G (TG) has been shown to significantly inhibit the early life-cycle phases of several respiratory viruses [37].

While most temporins are specifically active against Gram-positive bacteria, the isoform L (Temporin L, TL) (Phe-Val-Gln-Trp-Phe-Ser-Lys-Phe-Leu-Gly-Arg-Ile-Leu-NH2), is endowed of great efficiency not only against Gram-positive bacteria but also against Gram-negative bacteria and yeast strains [38]. On the other hand, while most temporins exert minor toxicity, especially against human erythrocytes at microbicidal concentrations, TL has a higher level of cytotoxicity [39]. Previous studies identified a direct correlation between TL hemolytic activity and its α-helical content [40]. By reducing the helicity percentage, it was possible to increase the therapeutic index of the peptide and to maintain its antimicrobial effectiveness against both bacteria and yeasts [40,41].

Considering the broad-range activity of TL as an antibacterial, we hypothesized that TL could also exert antiviral activity. It is, in fact, unlikely that frogs and other vertebrates would have developed a conserved and finely tuned mechanism in which a single peptide either works alone or is toxic to only one type of microorganism. The antimicrobial activity by peptides from amphibian skin, as an innate mechanism of defense, is unlikely to be caused by a single peptide but a combination of various peptides working in concert. This needs to be paralleled by the possible activity of each peptide on various targets [42,43]. Since, to date, TL antiviral properties have not been evaluated, we analysed the antiviral potential of TL and a set of peptidomimetic analogues against a large set of viruses comprising enveloped, naked, DNA and RNA viruses. Our results showed potent and selective antiviral activity of peptides derived from TL. This is the basis for the development of novel temporin-based anti-infective drugs to be used in the context of the arising drug resistance and epidemic and pandemic emergencies.

## 2. Results

### 2.1. Native Temporin L and Its Analog ([Pro3, DLeu9]TL) Antiviral Activities

The antiviral effect of the TL peptide was investigated in vitro against several viral pathogens that may severely threaten human health. Several enveloped viruses were used in this study including the enveloped herpes simplex viruses (HSV-1 and HSV-2), as examples of DNA viruses, and the human coronaviruses (HCoV-229E, HCoV-OC43 and SARS-CoV-2), MeV, HPIV-3 and influenza virus (subtype H1N1), as well as nonenveloped viruses such as poliovirus (Sb-1) and CV-B3. As described in the introduction, most temporins have been considered primarily effective in the inhibition of Gram-positive bacterial growth within concentrations of 2.5 and 50 μM [44]. The exceptions are represented by temporin DRa [45] and TL [38,39], which are also active against Gram-negative bacteria. Gram-negative bacteria present an outer membrane surrounding the peptidoglycan shell, and despite the peculiar differences between outer membranes (OMs) and other biological membranes, our reasoning was that these two particular temporins may be more effective against lipid membranes, and therefore able to affect enveloped viruses. Therefore, in the effort to identify a real broad-spectrum lead compound that could be effective also against viruses, besides microbes, we focused our interest on TL. In comparison to other temporins, TL is also known for its disruptive hemolytic activity expressed in a high level of toxicity for eukaryotic membranes. Nevertheless, a strong correlation between the hemolytic activity and the α-helix propensity has been shown in recent studies evaluating the structure-activity relationships of TL and a set of synthetic analogues [40]. An interesting compound based on TL was identified, [Pro^3^, DLeu^9^]TL, showing conserved antibacterial activity and a highly reduced cytotoxic effect in vitro. In this compound, the concurrent substitution of glutamine in position 3 (Gln^3^) and leucine in position 9 (Leu^9^) with proline and a D-enantiomer (DLeu), respectively, determined the advantageous consequence of maintaining a considerable inhibitory effect against both Gram-positive and Gram-negative bacteria, with an indicative reduction of toxicity, by disrupting the α-helical content of TL [46].

The two peptides selected in the present study were TL and its less toxic analogue [Pro^3^, DLeu^9^]TL (from now on named TL1, as described in Table 1).

**Figure 1:**
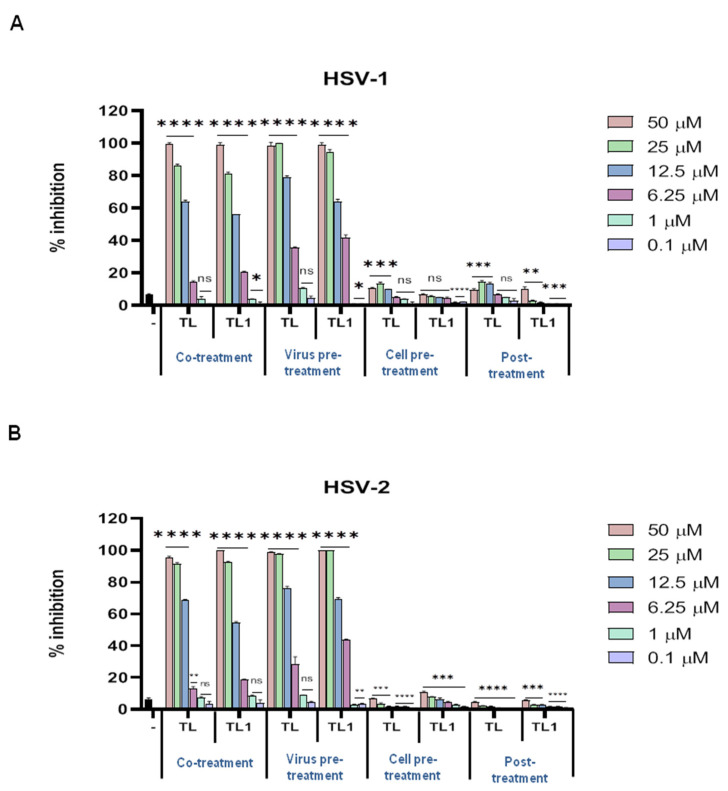
Antiviral activity of TL and TL1 against *Herpesviridae* members. Peptides were added in four different “time-of-addition” experiments and their antiviral potential was analysed against (**A**) HSV-1 and (**B**) HSV-2. **** *p* < 0.0001; *** *p* < 0.001; ** *p* < 0.01; * *p* < 0.1; ns: nonsignificant. – indicates the infected and not treated cells.

In our initial topical screening assays, we focused on the *Herpesviridae* family. Both HSV-1 and HSV-2 were mixed with different concentrations (from 0.1 μM to 50 μM) of TL and TL1 and were directly added to the Vero cells in the experiment named co-treatment assay. Peptide-free controls (virus plus cell culture medium only) were inoculated in parallel on cell monolayers. After 2 h of incubation at 37 °C to allow virus adsorption and penetration, the mixture (virus/peptide) was removed by washing with PBS and the plates were left at 37 °C in 5% CO_2_ after the addition of CMC for 48 h. After incubation, plaques were scored for measuring the amount of inhibition obtained compared to the peptide-free control. The results showed that both TL and TL1 were able to inhibit infectivity of the two members of the *alphaherpesvirinae* subfamily in a dose-dependent manner (Figure 1).

**Figure 2:**
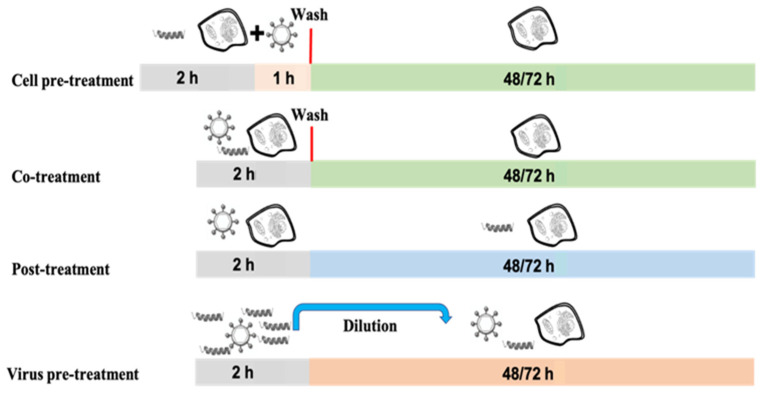
Four experimental schemes to study the virus pretreatment and the cell pretreatment, co-treatment, and post treatment effects of the TL-based peptides on viral infectivity. For cell pretreatment, each peptide was incubated with cells for 2 h, media was then removed, and virus inoculum added and incubated for 60 min, then inoculum was removed and replaced with fresh medium and cells were incubated. For virus pretreatment, the virus was incubated with peptide for 2 h then diluted to obtain ineffective peptide concentrations and added to cells. Cotreatment wells received compound and virus inoculum simultaneously, incubated for 2 h, then media was replaced, and cells were incubated. Post treatment wells were infected with virus for 2 h followed by inoculum removal and replacement with peptides in the media. In each experiment cells were incubated with fresh medium containing CMC from 48 to 72 h (depending on the virus used) and plaques were scored.

To better understand the inhibitory effect of TL and TL1 on the propagation of HSV-1 and HSV-2, we examined whether the peptides directly damaged virus particles or indirectly interacted with the host cells preinfection or postinfection. In practice, we performed three different “time-of-addition” experiments, namely (1) virus pretreatment, (2) cell pretreatment, and (3) cell post treatment experimental conditions, to elucidate the antiviral mechanisms of action of these two peptides. To find out whether TL and TL1 peptides exerted their inhibitory activity by interacting directly with HSV virions, we performed the virus pretreatment assay. Instead of simultaneously adding peptides and virus to the cells, viral particles were pretreated with peptides for 2 h at 37 °C. After this preincubation, the virus-peptide mixture was diluted so that the peptides were in a nonactive concentration and the virus MOI was 0.01 pfu/cell. After incubation for 48 h, residual infectivity was measured by plaque scoring. The two peptides showed strong inhibition of viral infectivity and were active at similar relative concentrations against both viruses (Figure 1). Subsequently, further experiments were conducted to determine whether the peptides could function at post-entry into cells (curative) or preinfection (prophylactic) stages (Figure 2).

Vero cell monolayers were treated with peptides for 2 h before virus addition (cell pre-treatment assay) or after (post treatment assay) virus penetration into cells to assess the activity of peptides in a post entry stage of the virus replicative cycle. No or minor inhibitory effect of the two peptides on both HSV-1 and HSV-2 could be detected (the obtained data are shown in Figure 1). This implied that these peptides probably inhibited herpesviruses infection mainly by directly interacting with viral particles. The 50% inhibitory concentrations (IC_50_) were 8.55 μM and 9.99 μM for HSV-1, and of 8.28 μM and 8.86 μM for HSV-2 vs. TL and TL1, respectively (all IC_50_ were calculated for virus pretreatment assay). The 90% inhibitory concentrations (IC_90_) were 15.66 μM and 18.69 μM for HSV-1, and 16.04 μM and 16.71 μM for HSV-2 vs. TL and TL1, respectively.

**Figure 3:**
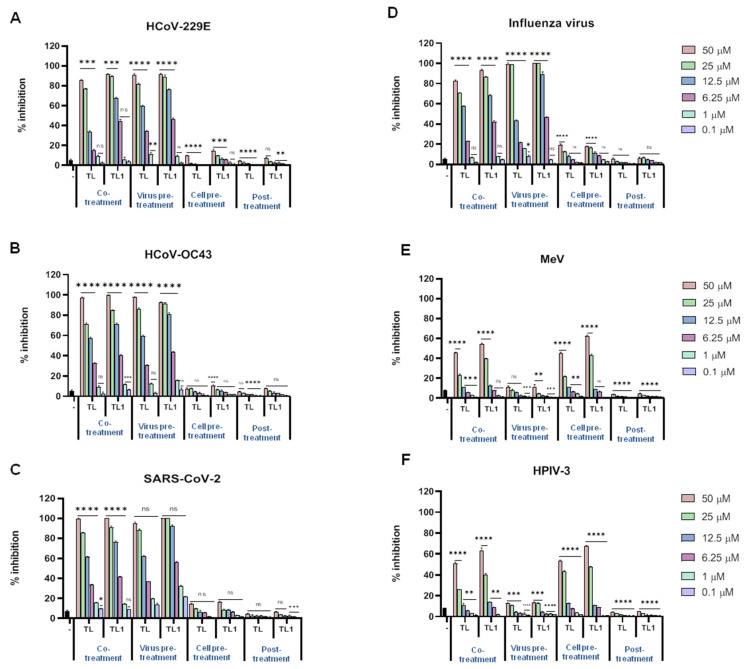
Antiviral activity of TL and TL1 against enveloped RNA virus. Peptides were added in four different “time-of-addition” experiments and their antiviral potential has been analysed against (**A**) HCoV-229E; (**B**) HCoV-OC43; (**C**) SARS-CoV-2; (**D**) Influenza virus; (**E**) MeV; and (**F**) HPIV-3. **** *p* < 0.0001; *** *p* < 0.001; ** *p* < 0.01; * *p* < 0.1; ns: nonsignificant. – indicates the infected and not treated cells.

To further characterize the inhibitory effect of TL and TL1, in vitro infection inhibition assays were performed with a collection of enveloped viruses that differ in genome content compared to herpesviruses (RNA versus DNA), namely: HCoV-229E, HCoV-OC43 and SARS-CoV-2, MeV, HPIV-3 and influenza virus (subtype H1N1) (Figure 3).

Four different times of addition experiments were performed, and the results showed a clear inhibition of infectivity in the cotreatment assay and in the virus pretreatment assay, while a minor efficiency was reported for the remaining two time-of-addition assays. Surprisingly, this was not the case for both paramyxoviruses analysed in the present study (Figure 3E,F), but possible differences in the lipid composition of their envelopes can possibly explain such a different behavior. MeV and HPIV-3 were not affected by both TL and TL1 in the virus pretreatment assay, and only minor evidence for a reduction of infectivity was found in the cotreatment assay. On the contrary, when the cell pretreatment assay was performed, a reduction of infectivity of over 40% with both peptides at the concentration of 25 μM was observed. No activity was recorded in the post treatment assay against tested paramyxoviruses.

Next, the effect of TL and TL1 on the infection of two nonenveloped viruses was tested. The infectivity of poliovirus Sb-1 and CV-B3 viruses was not altered in the presence of both peptides, as measured by plaque reduction assays following the four described experimental settings (Supplementary Figure S1). These results identify the TL and TL1 peptides as broad-spectrum antiviral peptides acting on enveloped viruses.

### 2.2. Cytotoxicity of Native Temporin L and Its Analog ([Pro3, DLeu9]TL)

We examined the effects of TL and TL1 on cell viability after incubating Vero cells with different concentrations of each peptide for 2 h, resembling the various treatments of cells used in our experimental protocols, and 24 h as a long exposure indicator. Cytotoxicity was evaluated monitoring cell viability using the 3-(4.5-dimethylthiazol-2-yl)-2.5-diphenyltetrazolium bromide (MTT) assay and expressed as percentage of inhibition of MTT reduction to its insoluble formazan crystals by mitochondrial dehydrogenases, compared to the untreated control cells (Supplementary Figure S2A,B). The nonlinear regression analysis result indicated that the 50% cytotoxic concentration (CC_50_) of TL was 19.61 μM and the CC_50_ of TL1 was 42.18 μM (all CC_50_ were calculated after 2 h of peptide treatment). Because some direct-acting antivirals disturb the envelope structure, they may also damage the cell membrane at similar concentrations, making their use hazardous. Mature mammalian red blood cells have no nucleus and low cell membrane repair ability. To provide further evidence to support the development of TL and its analog as potential therapeutics, we evaluated their ability to cause hemolysis of red blood cells. The results of the hemolytic activity shown in Supplementary Figure S2C indicated that both TL and TL1 were practically devoid of hemolytic activity at their antiviral concentrations, showing residual hemolytic activity only at concentrations equal or above 50 μM. At lower concentrations, a consistent lower hemolysis was recorded for TL1 compared to the parent peptide TL. This result is similar to the cytotoxicity analysed in Vero cells, where cell viability is higher when using TL1.

When toxicity data were evaluated in conjunction with antiviral results, we observed that TL1 retained appreciable levels of inhibitory activity against the virus infections along with moderate cytotoxicity; therefore, TL1 represents a potentially useful lead compound for further exploitation.

### 2.3. Cytotoxicity and Antiviral Activities of Gly10-Replaced TL1 Analogues

With a potential lead peptide (TL1) endowed with a specific antiviral activity in hand, we decided to investigate by single-point modification the possibility to improve its antiviral efficacy and further contain the low cytolytic features. In particular, we synthesized a set of derivatives, previously analysed in antibacterial assays [48]. The glycine (Gly) in position 10 was replaced with appropriate amino acids characterized by (i) the propensity to disrupt helicity (Pro, hydroxyproline (Hyp) and an unconventional amino acid, 2-aminoindane-2-carboxylic acid (Aic)); (ii) a positive charge or an indole ring in their side chain (Lys and Trp, respectively); and (iii) hydrophobic side chain [norleucine (Nle)]. In all these residues, both L and D isomers were used (except for the non-chiral Aic), as described in Table 2.

**Figure 4:**
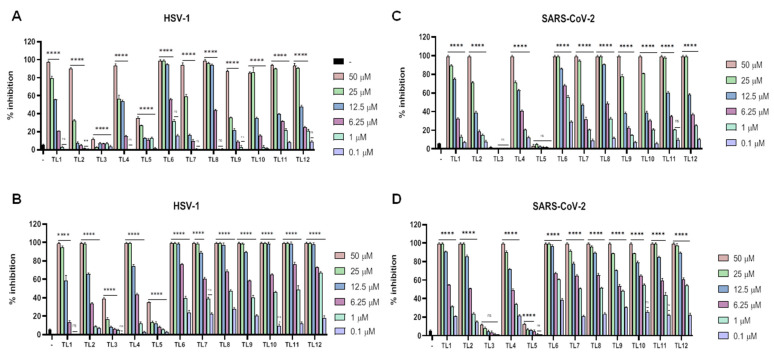
Antiviral activity of TL1 analogues against enveloped DNA and RNA viruses. (**A**,**C**) Co-treatment assay against HSV-1 and SARS-CoV-2 respectively; (**B**,**D**) virus pretreatment assay against HSV-1 and SARS-CoV-2 respectively. **** *p* < 0.0001; ** *p* < 0.01; ns: nonsignificant. – indicates the infected and not treated cells.

Cell viability was measured for TL1 analogues at both 2 h and 24 h using the MTT assay. The reported results (Supplementary Figure S3A,B) are consistent with previous data previously obtained [48]. Some minor divergences were probably due to the different cell lines used between the two studies, but in the present experimental model we preferred to use Vero cells, since these are generally employed for most of the antiviral assays. As depicted in Supplementary Figure S3A, the MTT assay after 2 h incubation showed that TL2, TL3, TL4, TL5, and TL6 had lower toxicity profiles compared to the parent peptide, especially at the higher concentration of 100 and 50 μM. When considering the window of possible therapeutic concentrations (below 25 μM), TL9 and TL10 presented good toxicity profiles with over 65% of viable cells. These profiles were conserved when the cytotoxicity assay was extended to 24 h, and confirmed by hemolysis assay (Supplementary Figure S3C). In consideration of the fact that TL1 was only active against enveloped viruses, subsequent experiments of viral inhibition were only conducted against enveloped viruses and not against CV-B3 and poliovirus Sb-1. We first analysed the inhibitory effect of each TL1 analog against HSV-1 and SARS-CoV-2 (in consideration of the present pandemic). Results are shown in Figure 4A,B for HSV-1, and Figure 4C,D for SARS-CoV-2.

As for the parent peptide TL1, these analogs were able to inhibit viral infectivity in a dose-dependent manner. In detail, the analogs were effective when they were added simultaneously with viruses on cells, or when the viruses were pretreated with peptides, showing a strong propensity for a virucidal effect. The antiviral activity was negligible in the case of cell pretreatment and post treatment assays (data not shown).

Peptide TL2, in which the residue of Gly^10^ was substituted with a Pro residue, with the intention of reducing helicity of the C-terminal region of the parent peptide TL1, showed a similar activity of the parent peptide TL1 for both HSV-1 and SARS-CoV-2. By replacing Pro10 with the corresponding enantiomer DPro, generating TL3, a dramatic loss of antiviral activity was recorded for the two viruses with only a negligible effect at the highest doses in the virus pretreatment assay. TL4, presenting a Hyp residue in position 10 also showed a generally conserved pattern of antiviral activity like TL1, but again the use of the enantiomer DHyp abolished any antiviral activity. Replacing the residue of Gly^10^ with Nle (TL6), characterized by an aliphatic side chain, led to a consistent improvement of the antiviral activity for both HSV-1 and SARS-CoV-2 which could be greatly appreciated for the latter virus. When Nle was replaced with its enantiomer DNle, the resulting peptide TL7 was not affected in the antiviral activity; on the contrary, it was even more effective against HSV-1 and SARS-CoV-2 when compared to TL6. In the other cases observed, TL3 and TL5, the insertion of D amino acids at position 10 had a profound disrupting effect. Replacement of the Gly^10^ residue with a residue of Lys (TL8), which has a positively charged side chain, allowed a substantial improvement in the anti-SARS-CoV-2 activity and a conserved activity against HSV-1. As for the previous peptides (TL6-TL7), the switch to the D amino acid in TL9 was not able to produce a reduction in activity, suggesting that chirality is of minor importance compared to the characteristics of the side chains present in the key position 10 of the antiviral peptide. TL10 and TL11 were designed with a residue of Trp and DTrp in position 10, respectively, and showed good antiviral activity against the two viruses analysed. Finally, we analysed the behavior of TL12 that is characterized by the insertion in position 10 of an unconventional amino acid Aic, a dialkylglycine derivative devoid of chirality. TL12 displayed a strong antiviral effect against HSV-1 and SARS-CoV-2; however, it exhibited consistent cytotoxicity. At this point, the whole panel of enveloped viruses was tested and the results (IC_90_ and IC_50_), reported in Table 3, clearly show a strong activity of the TL peptides against enveloped viruses, except for viruses belonging to the *Paramyxoviridae* family. At least with the members of this family we could observe a minor activity of the peptides as virucidal agents, but some antiviral activity was reported in the cell pretreatment assay with both MeV and HPIV-3, indicating a difference in the entry mechanism of paramyxoviruses compared to other enveloped viruses (data not shown). In the virus pretreatment of MeV, peptides TL6, TL8, TL9, TL10, TL11, and TL12 at the highest concentration showed a consistent viral inhibition from 60 to 80% (data not shown). This inhibition dropped drastically at the lowering of the concentrations of the relative peptides. Among the members of the *Coronaviridae* family, HCoV-229E showed minor susceptibility to the action of the TL analogs, probably reflecting the fact that HCoV-229E represents a member of the alfa-coronaviruses while SARS-CoV-2 and HCoV-OC43 are members of the beta-coronaviruses. To identify the best analogs to be considered for further modification, it was important to establish that the antiviral activities observed could be useful at concentrations that were possible to achieve without inducing toxic effects to cells. Therefore, the relative effectiveness of TL analogs in inhibiting viral replication was compared to cell viability (CC_50_ value/EC_50_ value) to obtain a therapeutic index (TI) (Table 3, Table 4, Tables S1 and S2).

The TI clearly showed that the best peptides for a therapeutic exploitation were ranked as follows: TL6, TL1, TL4, TL8, and TL9. To further analyse the most suitable analogue, we also performed a hemolytic assay using the concentration defined by the IC_50_ for each peptide as shown in Supplementary Figure S3B. Considering all the obtained results, TL6 was selected as the most interesting analogue for further modification by the addition of lipidic tags in the attempt to intensify the antiviral peptide concentrations on membranes during inhibition experiments.

### 2.4. Cytotoxicity and Antiviral Activities of TL6 and Its Lipid-Conjugates

In order to exploit the potential and to enhance the peptides’ antiviral activity, we chose the TL analog with the best TI, namely TL6, to add a cholesterol tag at its N- or C-terminal side (Table 5).

Lipidation of AMPs has a documented impact on improving their antimicrobial and antiviral effectiveness. The lipid tail can facilitate peptide insertion in lipid membranes and/or induce a self-organization into micelles that could provide a multimeric display of active peptides. As a starting strategy, we analysed the effect of cholesterol attached at both sides (the N and C termini) and with a small spacer (constituted by a PEG4 linker and one cys or a cys linked to two additional gly residues). The first result of note for cholesterol-conjugated TL6 peptides was the observation of a considerable improvement of their safety profiles. CC_50_ values are shown in Supplementary Figure S4 and are consistently higher than the counterpart parent peptide TL6 without lipidation. In particular, we observed that after 2 h of treatment, almost no reduction of cell viability was induced. Only TL6.2, where the PEG4 has been attached to a double gly and a cys residues, showed a marginal toxicity of about 40% at concentration above 50 μM. When moving to a 24 h treatment, the toxicity slightly increased for the following peptides: TL6.1, TL6.2 and TL6.4. Peptide TL6.3 represented the less toxic peptide, although the level of toxicity was considered of minor importance for any of the cholesterol-conjugated TL6 peptides, since it was constitutively well below the concentration that proved to be effective as antiviral. In order to reduce the number of experiments performed, we decided to test our lipidized peptides against a subset of viruses that had shown to be of interest in the previous part of the present experimental work, namely, HSV-1 and SARS-CoV-2 (Table 6), and MeV and influenza virus (Table 7). Virus suspensions were mixed with different concentrations (from 0.1 μM to 50 μM) of cholesterol-conjugated TL6 peptides and were directly incubated with target cells in cotreatment assay as previously described. The results showed that all lipidized peptides were able to inhibit infectivity of the two viruses in a dose-dependent manner (data not shown) and with high efficiency in the low μM range, reaching almost 100% inhibition at the concentration of 12.5 μM or lower. Peptides exerted their activity also in the virus pretreatment assay, showing an increase of the inhibition of infectivity compared to TL6. In a similar fashion to the activity of TL6, cholesterol-conjugated TL6 peptides showed good efficiency in the cotreatment and virus pretreatment experiments, but it was interesting to note that the cell pretreatment assay showed a strong inhibitory activity of peptides, especially TL6.3 (data not shown). This is of interest, since the parent peptide was active to a minor extent in this assay only against MeV and HPIV-3 (both *Paramyxoviridae* members) but was ineffective against any other virus tested. The post treatment assay was ineffective (data not shown). The ability of cholesterol-conjugated TL6 peptides to augment their antiviral efficiency in the cotreatment and cell pretreatment assays is probably due to the hydrophobicity increment of TL6 and its facilitated incorporation into lipid bilayers to create areas of higher antiviral peptide density on the cell surface. On the other hand, a different mechanism could be the reason for incremented activity of the lipidized peptides in the virus pretreatment assays. The peptides could self-organize in larger structured molecules, such as micelles, and also induce secondary structures that may interfere more efficiently with viral envelopes. Of note was a marked increase of antiviral activity without the addition of further Gly residues at the site of attachment and also the stronger activity when cholesterol was added to the N-terminus of the TL6 peptide. The IC_50_ of TL6.3 was 0.89 μM for HSV-1, 0.76 μM for SARS-CoV-2, 22.3 μM for MeV, and 2.55 μM for influenza virus (Table 6 and Table 7).

The last objective of the present work was to investigate if different-length fatty acids provide the best modifications to enhance antiviral activity. Four fatty acids were selected: undecanoic acid (CH_3_(CH2)_9_CO), tridecanoic acid (CH_3_(CH2)_11_CO), pentadecanoic acid (CH_3_(CH2)_13_CO), and hexadecanoic acid (palmitic acid) (CH_3_(CH2)_14_CO). Considering that the peptides modified with cholesterol tags exerted the best antiviral activity when the cholesterol was attached to the N-terminus, these other fatty acids were attached to the same side. The fatty acids conjugated-TL6 peptides were tested in cytotoxicity assays and inhibition assays with the following viruses: HSV-1, SARS-CoV-2, MeV and influenza virus, and the results showed an increase of the activity compared to the parent peptide up to 50-fold (Table 6 and Table 7). The most active peptide was the one with the shortest fatty acid chain, namely undecanoic acid (TL6.5), while the antiviral activity decreased with elongation of the carbon chain, with the lowest inhibitory activity for palmitic acid. Fatty acids greatly reduced the level of toxicity as observed with cholesterol-conjugated TL6 peptides. Finally, in order to understand the feasibility of the use of our selected peptides as human antivirals we performed additional cytotoxicity assays on peripheral blood mononuclear cells (PBMCs). Five peptides were chosen as representatives and were selected according to their higher antiviral activity, namely TL, TL1, TL6, TL6.3 and TL6.5. Cytotoxicity was measured by MTT assays at two time points: 2 h and 24 h. Results (Supplementary Figure S4A,B) showed a minor damage on PMBC that was comparable to the data obtained on Vero cells.

## 3. Discussion

Outbreaks of severe pathogenic viral infections (Avian and swine influenza viruses, coronaviruses, Ebola virus, Zika virus, Lassa fever virus), have occurred in recent years, and many other viruses are still highly diffused and endemic in many places worldwide (HIV, herpesviruses, hepatitis viruses, diarrhea viruses, and many others). The most recent viral disease is pneumonia due to infection by the coronavirus SARS-CoV-2, which is currently circulating globally causing huge public health and economic problems. These outbreaks highlight the urgent need for new strategies and approaches to develop efficient antiviral drugs with broad-spectrum activities for prophylactic and therapeutic treatments. However, current antiviral efforts, usually based on biochemical principles, are mainly focused on one virus at a time, following the traditional “one bug-one drug” paradigm, and are very limited in the coverage of viruses they target. These single virus and single target strategies have also been hampered by the rapid mutagenic ability of many viruses, and changing viral antigenic specificity may easily create escape mutants resistant to the single target antiviral. Since the risk of future viral outbreaks will continue to grow everywhere, a broad-spectrum antiviral strategy seems better-suited for responding to an increasing diversity of highly pathogenic viruses in a timely and effective manner. Strikingly, over 85% of major viral epidemics and pandemics in the past decade have been caused by membrane-enveloped viruses, which share their mechanism of fusion with the host cell membrane. This fusion mechanism is generally triggered by the virus encoded glycoproteins present on the envelope of these viruses. Fusion proteins are classified in three different classes according to their structural and functional domains, and among them, class 1 fusion proteins (present on viruses such as HIV, Influenza, measles and other parainfluenza viruses, Ebola, coronaviruses and some others) have been extensively studied. A powerful strategy using peptides that bind to the forming 6-helix bundle has proven to be very efficacious in stopping viral infectivity at the early stages of infection. This strategy has recently been widely used against SARS-CoV-2 [49,50], but again it is strictly dependent on the specific virus (or at least virus family) and antigenic variability. On the other hand, several studies have also shown that some antiviral peptides derived from classical AMPs are active against a wide range of enveloped and nonenveloped viruses [51,52,53,54,55,56,57,58].

The putative mechanism of action of AVPs for exerting their antiviral activity seems to be: (i) blocking early steps of viral entry by surface carbohydrate interaction, (ii) blocking viral attachment or penetration into the host cells by interactions with specific cellular receptors, (iii) interaction and inactivation of viral envelope glycoproteins, (iv) modulation of host cell antiviral responses, and (v) blocking intracellular expression of viral genes and/or production of viral proteins. However, no unequivocal correlation between AVP structures and viral inhibition has so far become obvious. In fact, striking differences from peptide to peptide are generally observed. Mechanistically, many AVPs exhibit their virucidal actions by direct disruption of the outer surface membrane of the virus particle. Due to this unique membrane-targeting activity, AVPs may have the potential to control viral species that are resistant to currently used antiviral agents [43].

Searching for pan-antivirals acting as multitarget inhibitors has not yet been explored in a satisfactory manner. Therefore, we aimed at identifying a broad-spectrum antiviral peptide derived from TL with potent antiviral activity. A phenotypic-based study was performed and TL and TL1 showed a discrete potency in antiviral assays, and we confirmed minor toxicity of TL1 on mammalian cells and human erythrocytes. The fact that both peptides showed a greatly reduced or null activity against nonenveloped viruses, and also at prophylactic and curative stages, indicated that peptides need to act directly with the viruses and that the main activity of peptides was exerted against the viral membrane. Putative mechanisms could involve interference with the proper lipid bilayer organization of the envelope, interaction with viral glycoproteins, or merely a hindrance mechanism able to interfere with the attachment and fusion steps to occur. Moving on from the positive results of the antiviral activity of the TL1 analog, we proceeded to exploration of the role played by the Gly in position 10, as previously done for assessing antibacterial activity [48]. Eleven different analogues were produced and analysed. The amino acids chosen for substituting Gly10 were Pro, Hyp, and Aic to disrupt helicity, Lys for the positive charge in the side chain; Nle presenting a hydrophobic side chain and Trp with its indole ring. All substituted amino acids were inserted with their L and D configurations with the exception of the Aic residue (Table 2). Most of the TL analogs maintained their antiviral activity and there was not a significant difference when considering the different viruses used in the experimental models. In analogy with the results obtained with TL and TL1, the Gly10 analogs of TL1 were effective in virus pretreatment and cotreatment assays, confirming a preference for targeting the viral envelope before or at the moment of encountering cell membranes. They exhibited minor or null activity when first incubating cells with peptides, with the exception of *Paramixoviridae* members (MeV and HPIV-3). The most interesting result is represented by peptide TL6, which has a Nle in substitution of the Gly10, increasing the hydrophobicity proved to increase antiviral activity and reduce cytotoxicity. Norleucine may be a key factor in driving peptide interactions with membranes. Other peptides with enhanced antiviral activity were TL8, TL10, TL11 and TL12, but the latter two also had highly accentuated toxicity both at 2 h and 24 h post-stimulation. The hemolytic activity was considerable compared to their template TL1, and especially compared to TL4, TL6, TL7 and TL9, which showed a clear increment of intact erythrocytes. Collectively, we observed that D enantiomers were much less active in any of the antiviral assays performed, even though a better toxicity profile was shown, except for TL11 with the DTrp in place of Gly10. The helicity of the peptide seems to be of uttermost importance since the three substitutions (Pro, Hyp, Aic), disrupting the helix, rendered the peptides inactive against all the viruses tested, except for the Aic insertion, but at detriment of the toxicity profile and TI. Assuming that the parent peptide TL1 exerted its antiviral activity by disrupting viral membranes, the poor antiviral activity of Pro and Hyp substitution may be attributed to the detrimental effect of the mutations on inducing α-helical conformation on interaction with the viral membranes. On the other hand, the insertion of Lys and Trp with a positive charge in the side chain and an indole ring, respectively, produced peptides with enhanced antiviral capacity but substantially increased toxicity; therefore, with poor TIs. Collectively, our results show that the effect of the peptide with the best TI, namely TL6, is mainly due to its direct interaction and damage of viral membranes. Damage to viral membranes could be similar to damage to cellular membranes, but a series of considerations can be drawn to explain the reduced toxicity. The lipids of enveloped viruses are derived from their host cell membrane, but the lipid composition often differs between cell membranes and viral envelopes. Indeed, some viruses are enriched with specific lipids. For example, the influenza A membrane is cholesterol-rich, while the dengue membrane lacks cholesterol, and the HIV envelope contains negatively charged phosphatidylserine (PS) [59]. L compositions can play important roles in the membrane/envelope properties, such as stiffness, fluidity, and line tension, leading to different membrane interactions with TL peptides. As a matter of fact, during the fusion step of enveloped virus penetration, a key structure is represented [60,61,62,63] by the formation of the lipid stem, requiring a shift of the outer leaflet of the bilayer from a positive to a negative curvature. Therefore, a limitation of possible movements forced by TL peptides, which may stabilize the positive curvature, is expected to reduce the possibility of membrane fusion, leading to putative broad-spectrum antivirals with low cytotoxicity and reduced ability to select for resistance. Furthermore, the eukaryotic cell membrane is continuously recycling itself, with a high degree of self-renewal upon injury, while the lipid membrane loses this faculty once surrounding the viral nucleocapsid, becoming particularly prone to membrane damage. Extensive membrane damage may also hamper virus fusion with the host cell membrane by impacting the fluidity and curvatures of lipid membranes [64]. Finally, the curvature of the envelope itself is different compared to the curvature of the cell membrane, principally due to the relative sizes of viruses versus mammalian cells. TL peptides may be able to selectively induce pore formation in highly curved membrane structures (below ~250 nm in diameter), resulting in membrane lysis once a critical number of pores is formed, with the consequence of viral infectivity reduction [65].

To further exploit the results so far obtained, TL6 was further modified by adding a cholesterol tag at its N or C-terminal side. Several previous reports have described that by attaching a cholesterol group to a peptide fusion inhibitor the antiviral potency is productively augmented [66]. In general, it has been proven that cholesterol-tagging of peptides, derived from the coiled-coil C-terminal heptad repeats of viral fusion proteins of class 1 [64,66,67,68,69,70,71,72,73,74,75,76] produced infectivity inhibitors from 100 to 1000 more efficient than their parent peptides. Nevertheless, lipidation of AVPs, besides those from viral HR mimicking peptides, has not been deeply investigated yet. Our results are of interest since lipidation reduced toxicity of two logs and increased efficiency leading to infectivity reduction in the cell pretreatment assay. A main difference with known HR-derived lipidated peptides is that the most active peptide was TL6.3, which has the cholesterol tag attached to its N-terminal side, showing that the mechanism of action between the two different classes of entry inhibitors is profoundly different. When HR peptides interfere with the formation of the 6-helix bundle, their orientation from the cell membrane in which lipidated peptides are inserted is of uttermost importance, and C-terminal HR peptides show a better functionality when the lipid tag is attached to the C-terminus of the peptide. On the other hand, AVPs such as temporins may work in both orientations and probably in an almost planar position on the membrane’s surface. Therefore, a preference for the N-terminal attachment of the lipid tag has been reported. Moreover, the length of the lipidic tail and the length of the linker seem to be of minor importance. In fact, as opposed to HR-derived peptides, TL lipidated peptides preferred a shorter hydrocarbon tail and a shorter linker.

## 4. Materials and Methods

### 4.1. Chemistry

#### 4.1.1. Materials

N^α^-Fmoc-protected amino acids, Fmoc-Phe, Fmoc-Val, Fmoc-Pro and Fmoc-DPro, Fmoc-Trp(Boc) and Fmoc-DTrp(Boc), Fmoc-Ser(tBu), Fmoc-Lys(Boc) and Fmoc-DLys(Boc), Fmoc-Leu and Fmoc-DLeu, Fmoc-Gly, Fmoc-Arg(Pbf), Fmoc-Nle and Fmoc-DNle, all were purchased from GL Biochem Ltd. (Shanghai, China). Other unconventional N^α^-Fmoc-amino acids, namely Fmoc-Hyp(tBu) and Fmoc-DHyp(tBu), were purchased from Sigma-Aldrich (St. Louis, MO, USA), and Fmoc-Aic was acquired by Chem-Impex International (Wood Dale, IL, USA). Undecanoic, Tridecanoic, Pentadecanoic and Palmitic acids were purchased from Sigma-Aldrich. Cholesterol-PEG4 was obtained by following the synthetic procedures elsewhere reported [66]. Coupling reagents such as N,N,N′,N′-tetramethyl-O-(1H-benzo-triazol-1-yl)uronium hexafluorophosphate (HBTU) and 1-hydroxybenzotriazole (HOBt), as well as the Rink amide resin used, all were commercially obtained by GL Biochem Ltd. (Shanghai, China). N,N-diisopropylethylamine (DIEA), piperidine, and tri-fluoroacetic acid (TFA) were purchased from Iris-Biotech GMBH. Peptide synthesis solvents and reagents, such as N,N-dimethylformamide (DMF), dichloromethane (DCM), diethyl ether (Et2O), water and acetonitrile (MeCN) for HPLC, were reagent grade acquired from commercial sources (Sigma-Aldrich and VWR) and used without further purification.

#### 4.1.2. Peptide Synthesis

The synthesis of peptides TL1-TL12 was performed by using the ultrasound-assisted solid-phase peptide synthesis (US-SPPS) integrated with the Fmoc/tBu orthogonal protection strategy [77]. Each peptide was assembled on a Rink amide resin (0.1 mmol from 0.72 mmol/g as loading substitution) as the solid support to obtain amidated C-termini. The peptide assembly was performed by repeated cycles of Fmoc deprotection (20% piperidine in DMF, 0.5 + 1 min) and coupling reactions using Fmoc-aa (2 equiv), COMU (2 equiv), Oxyma (2 equiv), DIPEA (4 equiv), 5 min in DMF by ultrasonic irradiations.

#### 4.1.3. Conjugation of Cholesterol to TL6.1–TL6.4

The addition of Cholesterol-PEG4 was performed introducing a cysteine residue in C-terminus for TL6.1 and TL6.2 or in N-terminus for TL6.3 and TL6.4. The reaction between the bromoacetyl derivate of cholesterol-PEG4 and thiol group of cysteine was carried out as described elsewhere [78]. In particular, the peptide was dissolved in DMSO and Cholesterol-PEG4 reagent dissolved in THF was added by a syringe pump in the presence of DIPEA at room temperature overnight. The equivalents used were peptide/cholesterol-PEG/DIPEA in the ratio 1/1/2 mol/mol/mol and the product was purified by preparative HPLC using a linear gradient of MeCN (0.1% TFA) in water (0.1% TFA), from 10 to 90% over 30 min.

#### 4.1.4. Conjugation of Fatty Acids to TL6.5–TL6.8

The introduction of fatty acids in the N-terminus of peptides TL6.5–TL6.8 was accomplished as described elsewhere [79]. Briefly, the conjugation was carried out adding three equivalents of undecanoic (TL6.5), tridecanoic (TL6.6), pentadecanoic (TL6.7), and palmitic (TL6.8) acids, respectively, preactivated with HBTU/HOBt (three equivalents) as coupling/additive reagents in presence of DIEA (six equivalents) in DMF/DCM (1:1 *v*/*v*), by ultrasonic irradiation for 15 min. Upon filtering and washings of the resin with DMF and DCM, the addition of fatty acid was ascertained by Kaiser test and LC-MS analysis. Thus, peptides TL6.5–TL6.8 were released from the resin and simultaneously cleaved by their protecting groups by using a cocktail of TFA/TIS/H_2_O (95:2.5:2.5, *v*/*v*/*v*) at rt for 3 h. Finally, the resins were removed by filtration and crudes were recovered by precipitation with cool anhydrous diethyl ether as amorphous solids. The peptides were purified by RP-HPLC (Shimadzu Preparative Liquid Chromatograph LC-8A) equipped with a preparative column (Phenomenex Kinetex C18 column, 5 mm, 100 Å, 150 21.2 mm) using linear gradients of MeCN (0.1% TFA) in water (0.1% TFA), from 10 to 90% over 30 min, with a flow rate of 10 mL/min and UV detection at 220 nm.

### 4.2. Biology

#### 4.2.1. Cell and Virus Culture

Vero cells (ATCC CCL-81, Manassas, VA, USA) and Vero/hSLAM cells (ECACC 04091501, Porton Down, UK) and Madin Darby canine kidney (MDCK, CCL-34) were grown in Dulbecco’s Modified Eagle Medium (DMEM) with 4.5 g/L glucose (Microtech, Naples, Italy) supplemented with 100 IU/mL penicillin and 100 μg/mL streptomycin (Himedia, Naples, Italy) and 10% Fetal Bovine Serum (Microtech). PBMCs were isolated as follow. Buffy coats acquired from a healthy anonymous donor were used to isolate PBMCs on a Ficoll-Hypaque density gradient (Sigma-Aldrich) according to standard procedures. Then, PBMCs were grown (5 × 10^6^/mL) in RPMI 1640 (Microtech) supplemented with 10% FBS, 60 IU/mL of interleukin-2 (IL-2, Sigma-Aldrich) and 2 µg/mL of phytohemagglutinin (PHA) (Sigma-Aldrich).

HSV-1 (strain SC16), containing a lacZ gene driven by the cytomegalovirus IE-1 promoter to express beta-galactosidase, and HSV-2 (strain G, ATCC VR-734) were propagated on Vero cells, as previously reported. Measles virus (MeV, ATCC VR-24) was grown on VERO/hSLAM cells, while human parainfluenza virus 3 (HPIV-2, ATCC VR-92), HCoV-229E (ATCC VR-740), HCoV-OC43 (ATCC VR-1558) and SARS-CoV-2 (strain VR PV10734, kindly donated by the Lazzaro Spallanzani Hospital of Rome, Italy), Enterovirus B (coxsackievirus B3, ATCC VR-688) and Enterovirus C (Sb-1, poliovirus Sabin strain chat, ATCC VR-1562) were propagated on a Vero cell line, while influenza virus (strain H1N1, VR-1894) was propagated on MDKC cells, as reported elsewhere [80]. The original stock titers (pfu/mL) of the viruses used in this study were 10^9^ plaque-forming unit (pfu)/mL for HSV-1, HSV-2, and HCoV-229E, 10^8^ pfu/mL for measles virus, HPIV-2, coxsackievirus B3 (CV-B3) and poliovirus, and 10^7^ pfu/mL for HCoV-OC43, SARS-CoV-2 and influenza virus.

#### 4.2.2. Cytotoxicity

Vero cells were seeded in 96-well microtiter tissue culture plates (5 × 10^3^ cells /well) and incubated for 24 h at 37 °C in 5% CO_2_. PBMCs were isolated and then cultured at a concentration of 1 × 10^5^ cells/well.

The cytotoxicity for both cell types was evaluated by the MTT (Sigma-Aldrich) assay based on the [81] reduction of the yellowish MTT to the insoluble and dark blue formazan by viable and metabolically active cells. Temporin-based peptides were tested at four different concentrations (0.1, 1, 6.25, 12.5, 25, 50 and 100 µM) after 2 and 24 h. At the end of incubation, 100 μL of an MTT solution (5 mg/mL) was added in each well and incubated for 3 h at 37 °C. The supernatant was discarded and 100 μL of DMSO 100% (Sigma Aldrich) was added (to dissolve the formazan salts) for 10 min with vigorous agitation at room temperature. Cytotoxicity was evaluated by spectrophotometric reading at 540 nm. The viability of Vero cells in each well was presented as a percentage of control cells. All experiments were performed in triplicate, and the means standard deviations are reported. Nonlinear regression analysis was performed using GraphPad Prism software (GraphPad Software, San Diego, CA, USA) to determine the CC_50_.

#### 4.2.3. Hemolytic Assays

The hemolytic activity of the peptides was determined using fresh human erythrocytes from healthy donors as reported previously [81]. Briefly, the blood was centrifuged, and the erythrocytes were washed three times with 0.9% NaCl. Peptides were added to the erythrocyte suspension (5% *v*/*v*), at a final concentration ranging from 0.1 to 100 μM in a final volume of 100 μL. The samples were incubated with agitation at 37 °C for 60 min. The release of hemoglobin was monitored by measuring the absorbance (Abs) of the supernatant at 540 nm. The control for zero hemolysis (blank) consisted of erythrocytes suspended in the presence of peptide solvent. Hypotonically lysed erythrocytes were used as a standard for 100% hemolysis. The percentage of hemolysis was calculated using the equation:

% hemolysis = [(Abs_sample_ − Abs_blank_)/(Abs_total lysis_ − Abs_blank_)] × 100

All experiments were performed in triplicate, and the standard deviations are reported.

#### 4.2.4. Antiviral Activity

The antiviral activity of the peptides was evaluated through four different assays: cotreatment, virus pretreatment, cell pretreatment and post treatment assays, where the main difference is the timing of addition of TL and its analogues. Cells were plated in 12-well (2.5 × 10^5^ cells/well) and incubated for 24 h at 37 °C. In all assays, peptides were added to the medium without FBS and tested at noncytotoxic concentrations. All experiments were performed in triplicate. The inhibition rate of the infectivity was evaluated by plaque assay comparing the number of plaques obtained in the wells treated with the peptides to the plaques counted in positive control (cells infected with virus, without peptide). Cotreatment assay: Cells were treated simultaneously with virus at a multiplicity of infection (MOI) of 0.1 pfu/cell and the peptides at the concentrations described above for 2 h at 37 °C. Then the mixture (virus/compound) was removed and the complete medium (10% FBS), supplemented with carboxymethylcellulose at 5% (Sigma, C5678, C5013), was added and incubated for 48 h at 37 °C in 5% CO_2_. The cells were fixed with 4% formaldehyde (Sigma, F1635), stained with crystal violet 0.5% and the number of plaques scored. Virus pretreatment assay: Peptides were added to the virus (1 × 10^4^ pfu/mL) and incubated for 2 h at 37 °C. After incubation, each mixture (virus/peptide) was diluted so that the peptides were in a nonactive concentration and the virus was at MOI of 0.01 pfu/cell. The dilutions were added to cell monolayers for 1 h, and then the cells were incubated with CMC for 48 h. At the end, the cells were fixed, stained and the number of plaques scored. Cell pretreatment: Cells were pre-cooled at 4 °C for 30 min and, subsequently, the peptides were added and incubated for 2 h at 4 °C. Each virus was added to a MOI of 0.1 pfu/mL for 1 h at 37 °C. Finally, the cells were incubated with CMC for 48 h at 37 °C. Cells were fixed, stained and the number of plaques was scored. Post treatment: Cells were incubated with viruses (MOI 0.1 pfu/mL) for 2 h at 37 °C, after that the peptides were added and incubated with CMC for 48 h at 37 °C. The cells were then fixed, stained and the number of plaques scored. The experimental assay recording a higher antiviral activity for most of the viral models used was chosen for performing a non-linear regression analysis using GraphPad Prism software to determine the IC_50_ and IC_90_.

#### 4.2.5. Calculation of Therapeutic Index

The therapeutic index (TI) is a widely accepted parameter to represent the specificity of antimicrobial reagents. To evaluate the margin of safety that exists between the dose needed for antiviral effects and the dose that produces unwanted and possibly dangerous side effects (cytotoxicity), the TI for each peptide was tcalculated from the efficacy and cytotoxicity data (CC_50_/IC_50_). Larger values of TI indicate greater antiviral efficiency.

#### 4.2.6. Statistical Analysis

All tests were performed in triplicate and expressed as mean ± standard deviation (SD) calculated by GraphPad Prism (version 8.0.1). One-way ANOVA followed Dunnett’s multiple comparisons test was performed; a value of *p* ≤ 0.05 was considered significant. 

## 5. Conclusions

In summary, we have described a set of modifications of TL peptides aiming to maximize the effects of new compounds to mitigate infectivity of enveloped viruses. We found that these TL-modified peptides are able to inhibit a wide range of enveloped viruses through direct nonspecific interactions with viral surface components. Emerging and re-emerging virus outbreaks remind us of the urgent need for broad-spectrum antivirals; therefore, further studies, together with a rational optimization of lead compounds, able to inhibit both DNA and RNA enveloped viruses by preventing virus-host cell early interactions with minor induction of drug resistance, will provide us with a stronger armamentarium to face emerging virus outbreaks in future.

## Figures and Tables

**Table 1:** TL and [Pro^3^, DLeu^9^] TL. Names, codes, sequences, and some properties (molecular weight and helical percentage) of TL and [Pro^3^, DLeu^9^] TL peptides.

Name	Code	Sequence	MW (as TFA Salt)	%Helix in DPC *
Temporin L	TL	H-Phe-Val-Gln-Trp-Phe-Ser-Lys-Phe-Leu-Gly-Arg-Ile-Leu-NH_2_	1640.02	62.2
[Pro^3^, DLeu^9^] TL	TL1	H-Phe-Val-Pro-Trp-Phe-Ser-Lys-Phe-DLeu-Gly-Arg-Ile-Leu-NH_2_	1949.98	42

* See references [47,48].

**Table 2:** Names, codes, sequences, and some properties (molecular weight and helical percentage) of TL1 and Gly^10^-replaced TL1 analogues.

Name	Code	Sequence	MW (as TFA Salt)	%Helix in DPC *
[Pro^3^,DLeu^9^] TL	TL1	H-Phe-Val-Pro-Trp-Phe-Ser-Lys-Phe-DLeu-Gly-Arg-Ile-Leu-NH_2_	1949.98	42
[Pro^3^,DLeu^9^,Pro^10^] TL	TL2	H-Phe-Val-Pro-Trp-Phe-Ser-Lys-Phe-DLeu-Pro-Arg-Ile-Leu-NH_2_	1990.01	15
[Pro^3^,DLeu^9^,DPro^10^] TL	TL3	H-Phe-Val-Pro-Trp-Phe-Ser-Lys-Phe-DLeu-DPro-Arg-Ile-Leu-NH_2_	1990.01	8
[Pro^3^,DLeu^9^,Hyp^10^] TL	TL4	H-Phe-Val-Pro-Trp-Phe-Ser-Lys-Phe-DLeu-Hyp-Arg-Ile-Leu-NH_2_	2006.01	18
[Pro^3^,DLeu^9^,DHyp^10^] TL	TL5	H-Phe-Val-Pro-Trp-Phe-Ser-Lys-Phe-DLeu-DHyp-Arg-Ile-Leu-NH_2_	2006.01	9
[Pro^3^,DLeu^9^,Nle^10^] TL	TL6	H-Phe-Val-Pro-Trp-Phe-Ser-Lys-Phe-DLeu-Nle-Arg-Ile-Leu-NH_2_	2006.04	51
[Pro^3^,DLeu^9^,DNle^10^] TL	TL7	H-Phe-Val-Pro-Trp-Phe-Ser-Lys-Phe-DLeu-DNle-Arg-Ile-Leu-NH_2_	2006.04	30
[Pro^3^,DLeu^9^,Lys^10^] TL	TL8	H-Phe-Val-Pro-Trp-Phe-Ser-Lys-Phe-DLeu-Lys-Arg-Ile-Leu-NH_2_	2135.07	61
[Pro^3^,DLeu^9^,DLys^10^] TL	TL9	H-Phe-Val-Pro-Trp-Phe-Ser-Lys-Phe-DLeu-DLys-Arg-Ile-Leu-NH_2_	2079.04	30
[Pro^3^,DLeu^9^,Trp^10^] TL	TL10	H-Phe-Val-Pro-Trp-Phe-Ser-Lys-Phe-DLeu-Trp-Arg-Ile-Leu-NH_2_	2079.04	50
[Pro^3^,DLeu^9^,DTrp^10^] TL	TL11	H-Phe-Val-Pro-Trp-Phe-Ser-Lys-Phe-DLeu-DTrp-Arg-Ile-Leu-NH_2_	2079.04	40
[Pro^3^,DLeu^9^,Aic^10^] TL	TL12	H-Phe-Val-Pro-Trp-Phe-Ser-Lys-Phe-DLeu-Aic-Arg-Ile-Leu-NH_2_	2053.03	18

* See reference [48].

**Table 3:** CC_50_, IC_90_, IC_50_, and TI calculated for HSV-1 and SARS-CoV-2. Concentrations are expressed in μM.

	CC_50_	IC_90_		IC_50_		TI	
		HSV-1	SARS-CoV-2	HSV-1	SARS-CoV-2	HSV-1	SARS-CoV-2
TL1	42.18	18.69	12.14	9.99	4.62	4.22	9.13
TL2	56.52	18.76	13.98	7.70	4.82	7.34	11.73
TL3	>100.00	>50.00	>50.00	>50.00	>50.00	-	-
TL4	60.19	15.89	25.00	7.73	6.72	7.88	9.06
TL5	>100.00	>50.00	>50.00	>50.00	>50.00	-	-
TL6	64.52	9.12	12.15	2.66	0.53	24.26	121.74
TL7	32.16	12.83	17.41	3.65	1.00	8.81	32.16
TL8	22.32	10.84	12.93	2.49	0.88	8.96	25.36
TL9	45.92	12.71	25.33	3.53	4.63	13.01	9.92
TL10	35.47	8.25	27.71	3.01	0.88	11.78	40.31
TL11	24.12	7.89	14.85	1.86	4.07	12.97	5.93
TL12	8.28	1.58	13.85	0.68	0.65	12.18	12.74

**Table 4:** CC_50_, IC_90_, IC_50_ and TI calculated for measles (MeV) and influenza viruses. Concentrations are expressed in μM.

	CC_50_	IC_90_		IC_50_		TI	
		MeV	Influenza	MeV	Influenza	MeV	Influenza
TL1	42.18	>50.00	12.52	>50.00	6.74	-	6.26
TL2	56.52	>50.00	21.28	>50.00	11.95	-	4.73
TL3	>100.00	>50.00	>50.00	>50.00	>50.00	-	-
TL4	60.90	>50.00	31.65	>50.00	16.26	-	3.75
TL5	>100.00	>50.00	>50.00	>50.00	>50.00	-	-
TL6	64.52	>50.00	9.34	34.58	2.66	1.87	24.26
TL7	32.16	>50.00	13.49	>50.00	4.45	-	7.23
TL8	22.32	>50.00	9.78	37.10	3.40	0.60	6.56
TL9	45.92	>50.00	11.18	44.98	3.44	1.02	13.35
TL10	35.47	>50.00	12.09	42.82	3.79	0.83	9.36
TL11	24.12	>50.00	9.77	43.23	3.05	0.56	7.91
TL12	8.28	>50.00	13.88	30.42	0.71	0.27	11.66

**Table 5:** Names, codes, sequences, and molecular weight of TL6 and its lipid-conjugates.

Name	Code	Sequence	MW (as TFA Salt)
[Pro^3^,DLeu^9^,Nle^10^] TL	TL6	H-Phe-Val-Pro-Trp-Phe-Ser-Lys-Phe-DLeu-Nle-Arg-Ile-Leu-NH_2_	2006.04
[Pro^3^,DLeu^9^,Nle^10^] TL-C-CHOL	TL6.1	H-Phe-Val-Pro-Trp-Phe-Ser-Lys-Phe-DLeu-Nle-Arg-Ile-Leu-Cys-[PEG4-Cholesterol]-NH_2_	2708.10
[Pro^3^,DLeu^9^,Nle^10^] TL-GGC-CHOL	TL6.2	H-Phe-Val-Pro-Trp-Phe-Ser-Lys-Phe-DLeu-Nle-Arg-Ile-Leu-Gly-Gly-Cys-[PEG4-Cholesterol]-NH_2_	2858.24
CHOL-C-[Pro^3^,DLeu^9^,Nle^10^] TL	TL6.3	H-[Cholesterol-PEG4]-Cys-Phe-Val-Pro-Trp-Phe-Ser-Lys-Phe-DLeu-Nle-Arg-Ile-Leu-NH_2_	2708.10
CHOL-CGG-[Pro^3^,DLeu^9^,Nle^10^] TL	TL6.4	H-[Cholesterol-PEG4]-Cys-Gly-Gly-Phe-Val-Pro-Trp-Phe-Ser-Lys-Phe-DLeu-Nle-Arg-Ile-Leu-NH_2_	2858.24
Undecanoic-[Pro^3^,DLeu^9^,Nle^10^] TL	TL6.5	H-Undecanoic acid-Phe-Val-Pro-Trp-Phe-Ser-Lys-Phe-DLeu-Nle-Arg-Ile-Leu-NH_2_	2192.33
Tridecanoic-[Pro^3^,DLeu^9^,Nle^10^] TL	TL6.6	H-Tridecanoic acid-Phe-Val-Pro-Trp-Phe-Ser-Lys-Phe-DLeu-Nle-Arg-Ile-Leu-NH_2_	2220.39
Pentadecanoic-[Pro^3^,DLeu^9^,Nle^10^] TL	TL6.7	H-Pentadecanoic acid-Phe-Val-Pro-Trp-Phe-Ser-Lys-Phe-DLeu-Nle-Arg-Ile-Leu-NH_2_	2248.44
Hexadecenoic-[Pro^3^,DLeu^9^,Nle^10^] TL	TL6.8	H-Palmitic acid-Phe-Val-Pro-Trp-Phe-Ser-Lys-Phe-DLeu-Nle-Arg-Ile-Leu-NH_2_	2262.47

C_27_H_46_O: cholesterol, CH_3_(CH2)_9_CO: undecanoic acid moiety, CH_3_(CH2)_11_CO: tridecanoic acid moiety, CH_3_(CH2)_13_CO: pentadecanoic acid moiety, CH3(CH2)14CO: hexadecanoic acid moiety.

**Table 6:** CC_50_, IC_90_, IC_50_ and TI calculated for HSV-1 and SARS-CoV-2. Concentrations are expressed in μM.

	CC_50_	IC_90_		IC_50_		TI	
		HSV-1	SARS-CoV-2	HSV-1	SARS-CoV-2	HSV-1	SARS-CoV-2
TL6	64.5	9.12	12.15	2.66	0.53	24.25	121.70
TL6.1	>100.00	11.13	12.03	6.4	3.18	15.63	31.45
TL6.2	>100.00	47.95	49.95	9.54	12.12	10.48	8.25
TL6.3	>100.00	2.19	1.89	0.89	0.76	112.36	131.58
TL6.4	>100.00	8.21	10.21	2.32	1.02	43.10	98.04
TL6.5	>100.00	<0.10	<0.10	<0.10	<0.10	1000.00	1000.00
TL6.6	>100.00	2.86	5.86	1.39	0.39	71.94	256.41
TL6.7	>100.00	4.86	7.86	2.48	0.48	40.32	208.33
TL6.8	>100.00	4.96	6.96	3.13	0.77	31.95	129.87

**Table 7:** CC_50_, IC_90_, IC_50_ and TI calculated for Mev and influenza virus. Concentrations are expressed in μM.

	CC_50_	IC_90_		IC_50_		TI	
		MeV	Influenza	MeV	Influenza	MeV	Influenza
TL6	64.52	>50.00	9.12	34.58	2.66	1.87	24.26
TL6.1	>100.00	>50.00	10.02	39.1	5.55	2.56	18.02
TL6.2	>100.00	>50.00	49.90	40.89	21.12	2.45	4.73
TL6.3	>100.00	31.10	5.89	22.3	2.55	4.48	39.22
TL6.4	>100.00	49.90	9.90	28.88	6.02	3.46	16.61
TL6.5	>100.00	29.18	<0.10	10.01	<0.10	1000.00	1000.00
TL6.6	>100.00	>50.00	6.66	33.36	3.39	3.00	29.50
TL6.7	>100.00	>50.00	8.10	37.77	3.48	2.65	28.74
TL6.8	>100.00	>50.00	7.11	39.4	4.77	2.54	20.96

## Data Availability

The data presented in this study are available on request from the corresponding author. Authors can confirm that all relevant data are included in the article.

## Supplementary Materials

The following supporting information can be downloaded at: https://www.mdpi.com/article/10.3390/ijms23042060/s1.
