# Supplementary material for: Broad-Spectrum Antiviral Activity of the Amphibian Antimicrobial Peptide Temporin L and Its Analogs
Source: Int J Mol Sci. 2022 Feb 13;23(4):2060. doi: 10.3390/ijms23042060 (PMC8878748; doi:10.3390/ijms23042060)
Supplement: Supplementary file 1 [file ijms-23-02060-s001.zip › ijms-1559643-supplementary.pdf]

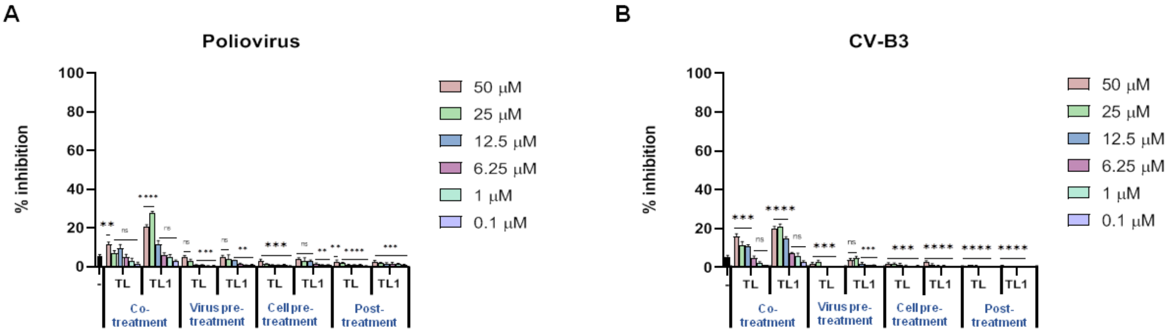

Figure S1. Antiviral activity of TL and TL1 against non enveloped RNA virus. Peptides were added in four different "time-of-addition" experiments and their antiviral potential has been analysed against (**A**) poliovirus and (**B**) CV-B3. **** $p < 0.0001$; *** $p < 0.001$; ** $p < 0.01$; ns: non-significant.

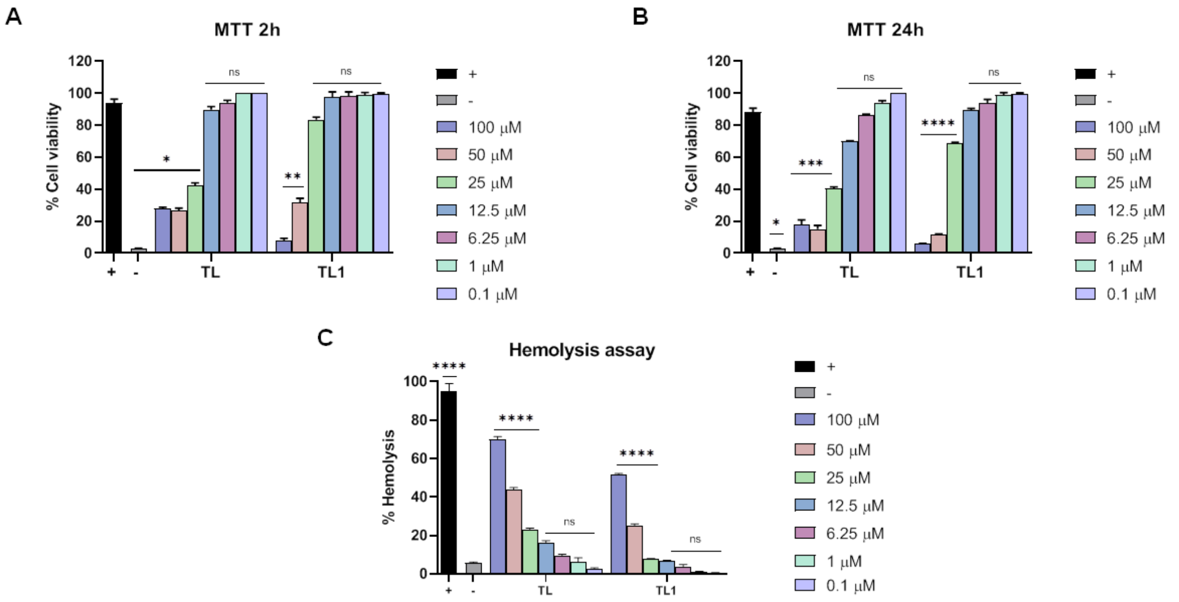

Figure S2. Peptide toxicity analysis on Vero cells. TL and TL1 toxicity was evaluated by MTT assay after (**A**) 2 hours and (**B**) 24 hours peptide treatment and (**C**) hemolysis assay. **** $p < 0.0001$; *** $p < 0.001$; ** $p < 0.01$, * $p < 0.1$; ns: non-significant.

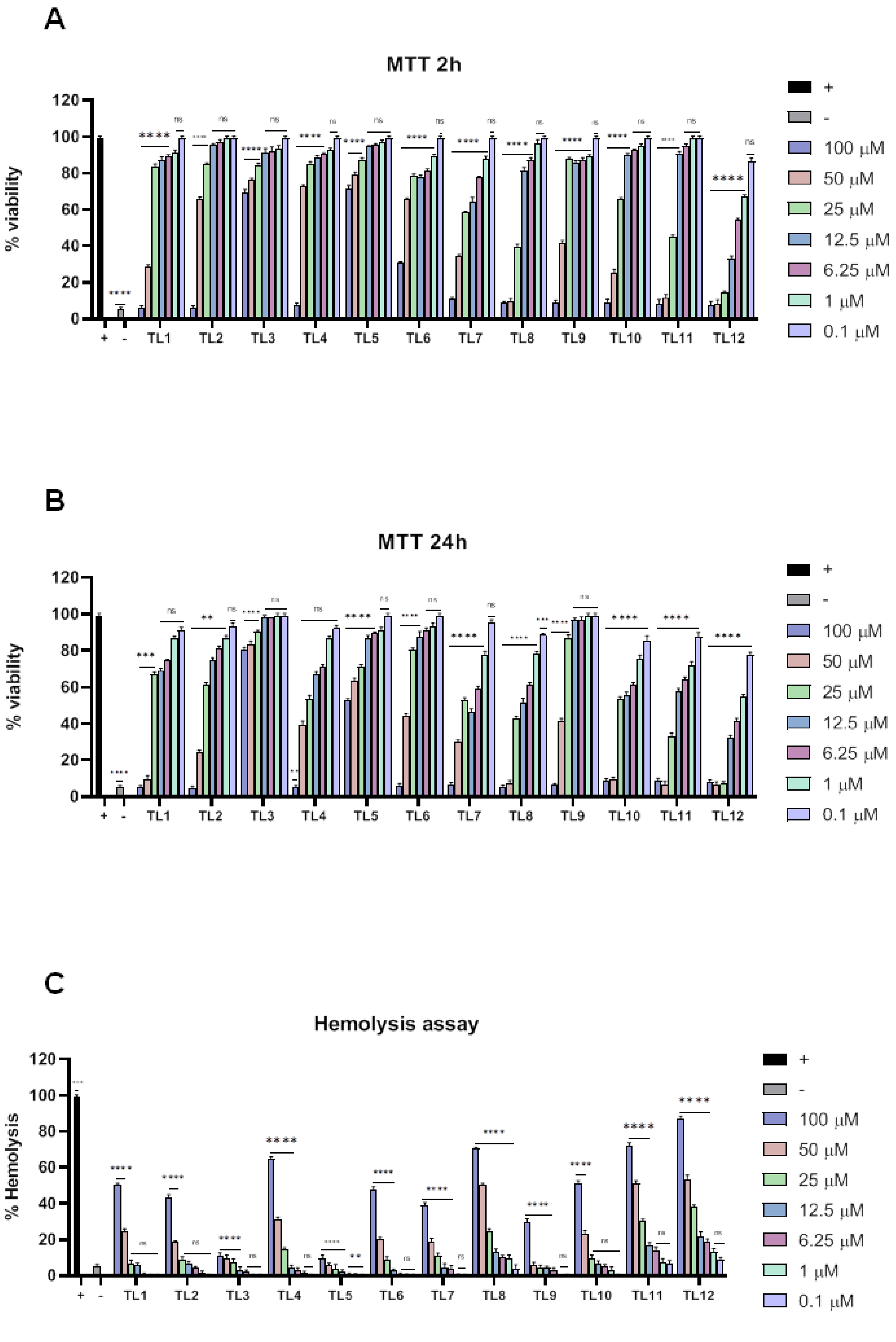

Figure S3. TL1 analogues toxicity analysis on Vero cells. Peptides toxicity was evaluated by MTT assay after (**A**) 2 hours and (**B**) 24 hours peptide treatment and (**C**) hemolysis assay. **** $p < 0.0001$; *** $p < 0.001$; ** $p < 0.01$; ns: non-significant.

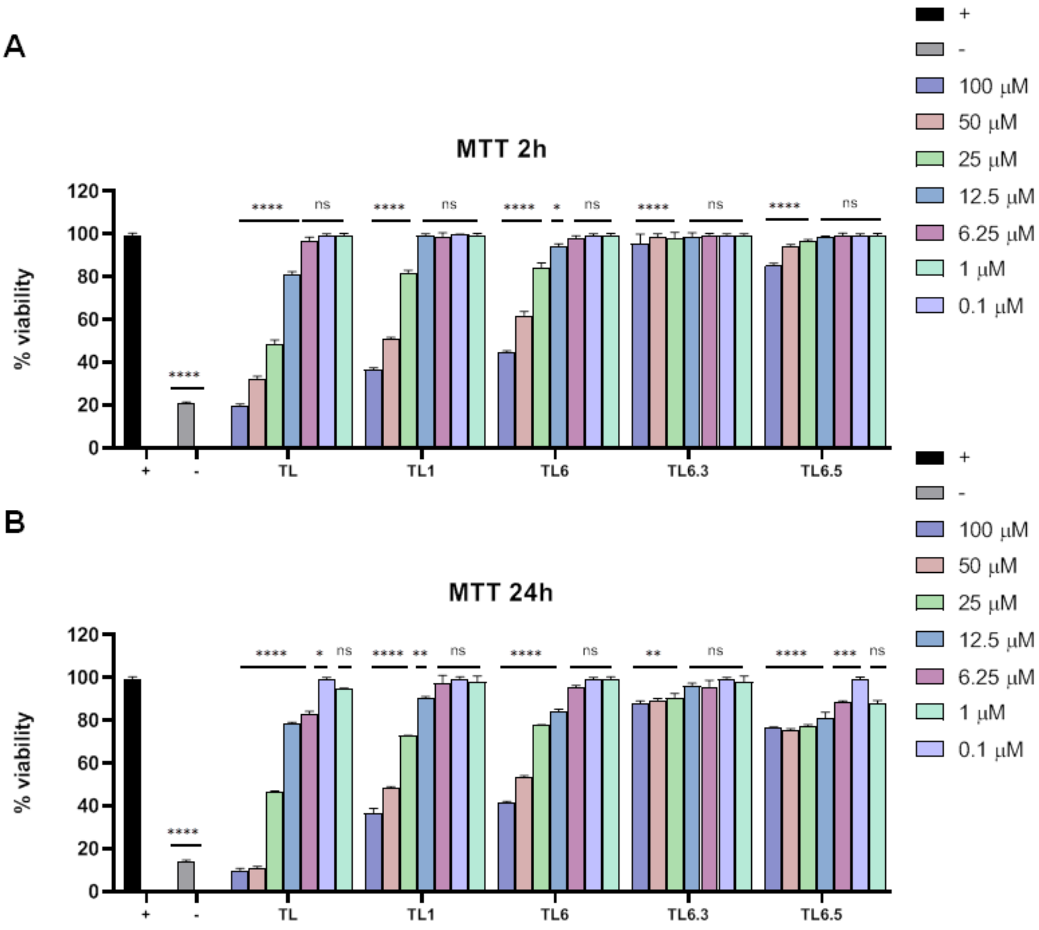

Figure S4. TL, TL1, TL6, TL6.3 and TL6.5 toxicity analysis on PBMCs. Peptides toxicity was evaluated by MTT assay after (**A**) 2 hours and (**B**) 24 hours peptide treatment. **** $p < 0.0001$; *** $p < 0.001$; ** $p < 0.01$, * $p < 0.1$; ns: non-significant.

Table S1. CC_{50}, IC_{90}, IC_{50} and TI calculated for HSV-2 and HPIV-3. Concentrations are expressed in µM.

	**CC_{50}**	**IC_{90}**		**IC_{50}**		**TI**	
		**HSV-2**	**HPIV-3**	**HSV-2**	**HPIV-3**	**HSV-2**	**HPIV-3**
**TL1**	42.18	16.71	>50.00	8.86	>50.00	4.76	-
**TL2**	56.52	19.20	>50.00	9.83	>50.00	5.75	-
**TL3**	>100.00	>50.00	>50.00	>50.00	>50.00	-	-
**TL4**	60.90	19.02	>50.00	9.61	>50.00	6.34	-
**TL5**	>100.00	>50.00	>50.00	>50.00	>50.00	-	-
**TL6**	64.52	6.28	>50.00	0.92	32.35	70.13	1.99
**TL7**	32.16	11.92	>50.00	3.13	>50.00	10.27	-
**TL8**	22.32	7.99	>50.00	1.00	40.69	22.32	0.55
**TL9**	45.92	12.50	>50.00	2.80	40.64	16.40	1.13
**TL10**	35.47	12.50	>50.00	0.77	>50.00	46.06	-
**TL11**	24.12	9.76	>50.00	0.58	41.18	41.59	0.59
**TL12**	8.28	11.68	>50.00	0.54	33.83	15.33	0.24

Table S2. CC_{50}, IC_{90}, IC_{50} and TI calculated for HCoV-229E and HCoV-OC43. Concentrations are expressed in μM.

	**CC_{50}**	**IC_{90}**		**IC_{50}**		**TI**	
		**HCoV-229E**	**HCoV-OC43**	**HCoV-229E**	**HCoV-OC43**	**HCoV-229E**	**HCoV-OC43**
**TL1**	42.18	30.07	14.98	7.66	7.33	5.51	5.75
**TL2**	56.52	50.00	24.13	10.56	11.31	5.35	5.00
**TL3**	>100.00	>50.00	>50.00	>50.00	>50.00	-	-
**TL4**	60.90	>50.00	27.65	11.74	11.33	5.19	5.38
**TL5**	>100.00	>50.00	>50.00	>50.00	>50.00	-	-
**TL6**	64.52	13.06	10.04	2.08	0.97	31.02	66.52
**TL7**	32.16	>50.00	24.74	6.21	8.55	5.18	3.76
**TL8**	22.32	50.00	16.30	4.39	2.90	5.08	7.70
**TL9**	45.92	50.00	29.17	5.39	8.29	8.52	5.54
**TL10**	35.47	50.00	24.17	0.91	0.96	38.98	36.95
**TL11**	24.12	50.00	17.41	0.92	5.10	26.22	4.73
**TL12**	8.28	20.90	13.18	0.74	0.62	11.19	13.35
